# Using T2-weighted magnetic resonance imaging-derived radiomics to classify cervical lymphadenopathy in children

**DOI:** 10.1007/s00247-024-05954-0

**Published:** 2024-06-27

**Authors:** Yanwen Xu, Caiting Chu, Qun Wang, Linjuan Xiang, Meina Lu, Weihui Yan, Lisu Huang

**Affiliations:** 1 Xinhua Hospital Affiliated to Shanghai Jiao Tong University School of Medicine, Shanghai, China; 2 Department of Radiology, Xinhua Hospital Affiliated to Shanghai Jiao Tong University School of Medicine, Shanghai, China; 3 Department of Infectious Diseases, Children’s Hospital, Zhejiang University School of Medicine, National Clinical Research Center for Child Health, 3333 Binsheng Road, Hangzhou, 310003 Zhejiang China; 4 Division of Pediatric Gastroenterology and Nutrition, Xinhua Hospital Affiliated to Shanghai Jiao Tong University School of Medicine, Shanghai, China

**Keywords:** Child, Histiocytic necrotizing lymphadenitis, Lymphadenopathy, Magnetic resonance imaging, Radiomics

## Abstract

**Background:**

Cervical lymphadenopathy is common in children and has diverse causes varying from benign to malignant, their similar manifestations making differential diagnosis difficult.

**Objective:**

This study aimed to investigate whether radiomic models using conventional magnetic resonance imaging (MRI) could classify pediatric cervical lymphadenopathy.

**Methods:**

A total of 419 cervical lymph nodes from 146 patients, and encompassing four common etiologies (Kikuchi disease, reactive hyperplasia, suppurative lymphadenitis and malignancy), were randomly divided into training and testing sets in a ratio of 7:3. For each lymph node, 1,218 features were extracted from T2-weighted images. Then, the least absolute shrinkage and selection operator (LASSO) models were used to select the most relevant ones. Two models were built using a support vector machine classifier, one was to classify benign and malignant lymph nodes and the other further distinguished four different diseases. The performance was assessed by receiver operating characteristic curves and decision curve analysis.

**Results:**

By LASSO, 20 features were selected to construct a model to distinguish benign and malignant lymph nodes, which achieved an area under the curve (AUC) of 0.89 and 0.80 in the training and testing sets, respectively. Sixteen features were selected to construct a model to distinguish four different cervical lymphadenopathies. For each etiology, Kikuchi disease, reactive hyperplasia, suppurative lymphadenitis, and malignancy, an AUC of 0.97, 0.91, 0.88, and 0.87 was achieved in the training set, and an AUC of 0.96, 0.80, 0.82, and 0.82 was achieved in the testing set, respectively.

**Conclusion:**

MRI-derived radiomic analysis provides a promising non-invasive approach for distinguishing causes of cervical lymphadenopathy in children.

**Graphical Abstract:**

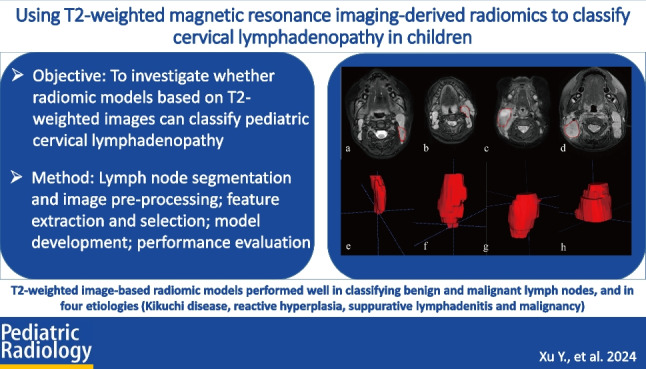

**Supplementary Information:**

The online version contains supplementary material available at 10.1007/s00247-024-05954-0.

## Introduction

Pediatric cervical lymphadenopathy is a common clinical finding with diverse causes varying from benign to malignant. The lymphatic system undergoes rapid development during childhood and reaches its peak at puberty. Consequently, the lymph nodes may enlarge under physiological conditions. It is reported that around 28% of healthy school children have palpable lymph nodes in the neck [1]. Furthermore, childhood is a stage at which respiratory tract infections are more likely to occur, and cervical lymph nodes enlarge in response to bacterial or viral infections [2, 3]. Notably, head and neck malignancies account for 12% of all pediatric malignancies [4]; common types include lymphoma, thyroid carcinoma, and metastatic nasopharyngeal carcinoma, with persistent enlargement of lymph nodes as an early warning sign [5, 6]. Kikuchi disease, also referred to as histiocytic necrotizing lymphadenitis, which shows a growing trend among Asian children, is also characterized by enlarged cervical lymph nodes and is a benign and self-limiting process, but is easily confused with lymphoma clinically and histologically [7].

However, the differential diagnosis of pediatric cervical lymphadenopathy is quite challenging due to their overlapping non-specific manifestations [2, 3], and pathological confirmation by biopsy is considered the gold standard. Currently, there is still no consensus on indication and timing of biopsy; parents are hesitant because of the invasive nature of the surgery, and their concerns include the impact general anesthesia has on children’s developing brain, risk of incision infection, and surgery costs.

For this reason, non-invasive imaging tools are becoming more and more valued. With the advantage of superior soft tissue contrast, multi-angle scanning, and no radiation damage, magnetic resonance imaging (MRI) might outperform ultrasound (US) and computed tomography (CT) to some extent. T2-weighted imaging is one of the essential components of neck MRI, and is useful for detecting pathology such as inflammation, abscesses, and tumors [8]. Some previous studies report that benign and malignant lymph nodes have different features on T2 sequences, but these features are still mainly morphological and the characterization of the internal structure is still limited [9, 10]. Radiomics offers an insight into the quantification of lesion characteristics that are imperceptible to human eyes but may be related to pathological changes. Recently, MRI-based radiomics models have been applied in the field of head and neck imaging, showing good performance in prediction of lymph node metastasis, extra-nodal extension status, and outcome prediction in patients with malignancy [11–15]. To our knowledge, benign cervical lymphadenopathy is not covered in current MRI-based radiomics models, and studies on pediatric patients are lacking.

Therefore, the aim of this study was to develop and validate radiomics models to classify cervical lymphadenopathy in children based on conventional axial T2 MRI scans.

## Materials and methods

### Study cohorts

This retrospective study was approved by the institutional ethics review board, and informed consent was waived. All patients met the following inclusion criteria: (1) aged <18 years; (2) underwent neck MRI examination because of cervical lymphadenopathy; (3) had enlarged lymph nodes with shortest diameter > 1.0 cm or largest diameter > 1.5 cm on axial images; (4) a histologically confirmed diagnosis. The exclusion criteria were as follows: (1) poor image quality due to apparent motion artifacts; (2) previous treatment (such as radiotherapy, chemotherapy, interventional therapy, or lymph node biopsy surgery). Through consecutive enrollment, we found the most common causes were reactive hyperplasia, suppurative lymphadenitis, Kikuchi disease, and malignancy; subsequently, disease groups with very few patients (<10) were eliminated. Ninety-two patients were recruited from Xinhua Hospital (affiliated to Shanghai Jiao Tong University School of Medicine) between January 2015 and August 2022 and 54 patients were recruited from Children’s Hospital, Zhejiang University School of Medicine between January 2017 and December 2022. The general clinical data of patients were collected from medical records.

### Equipment and post-processing

MRI data were acquired on three 3-tesla MR scanners (Siemens, Erlangen, Germany; Philips, Amsterdam, the Netherlands, and GE Medical System, Milwaukee, WI) using a 64-channel head and neck coil on axial T2-weighted sequences. Diffusion-weighted imaging sequences were performed in some patients, but these images were not included in the analyses due to limited numbers. A 4- to 5-mm slice thickness and matrix ≥175×260 were used. The scanning parameters are shown in Supplementary Material 1. To reduce the bias caused by the variability of imaging parameters and scan conditions of different MRI machines, all images were resampled into 1×1×1 mm^3^ and *z*-score normalization was used to eliminate the batch effect before radiomics feature extraction.

### Segmentation and feature extraction

**Fig. 1:**
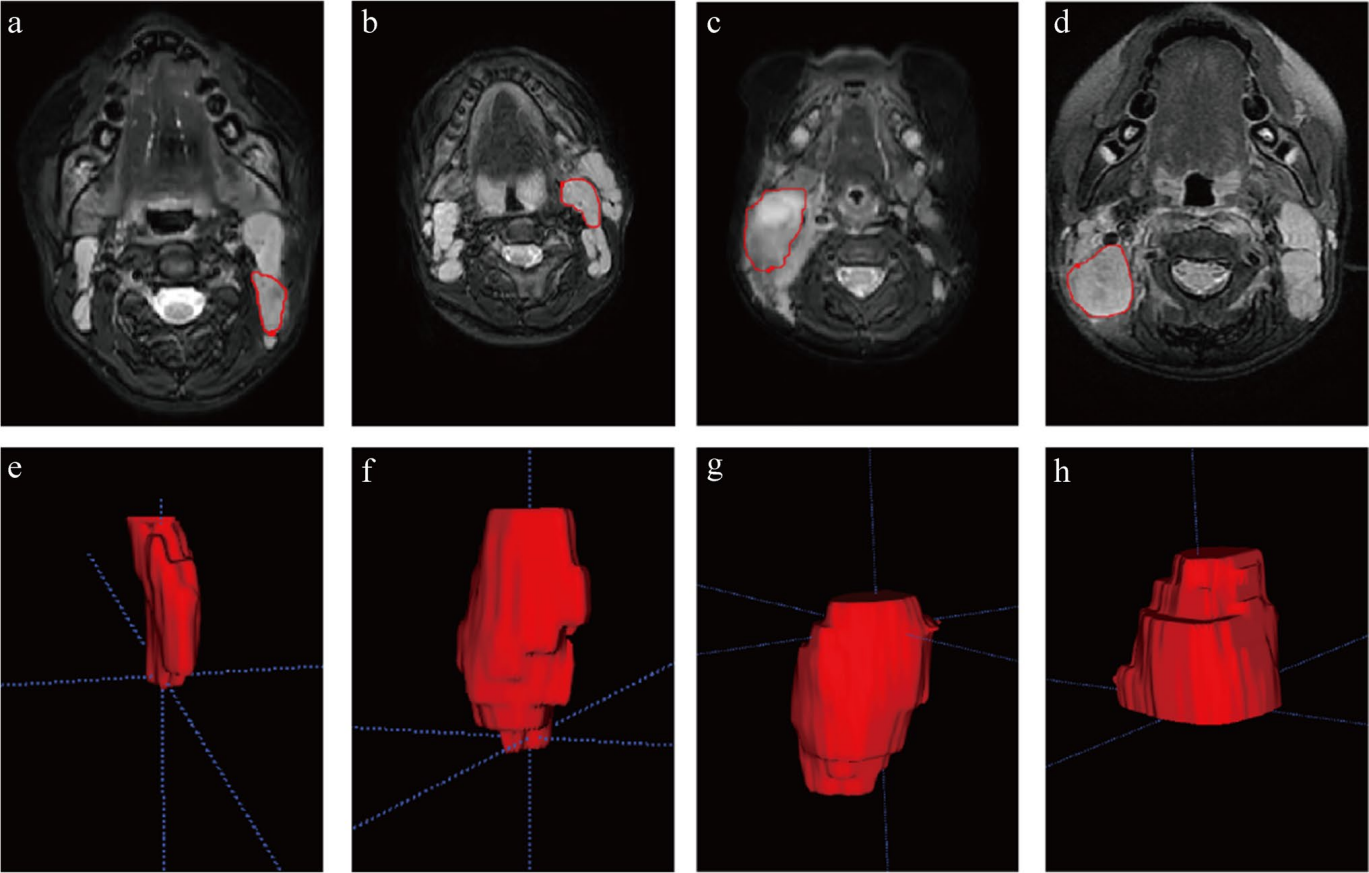
Axial T2 magnetic resonance scans show segmentation (*red*) of cervical lymphadenopathy (**a**–**d**) and 3-dimensional reconstructed images of the lymph nodes (**e**–**h**) in a 10-year-old girl with Kikuchi disease (**a**,** e**), an 11-year-old girl with reactive hyperplasia (**b**,** f**), a 2-year-old boy with suppurative lymphadenitis (**c**,** g**), and a 12-year-old boy with malignancy (**d**,** h**)

The volumes of interest (VOIs) were manually and independently segmented using ITK-SNAP (Version 3.8.0) by two radiologists Y.S. (a subspecialist in head and neck tumors with 2 years of experience) and C.C. (a pediatric infectious diseases subspecialist with 8 years of experience) who were blinded to pathologic diagnosis and clinical information. If the lymph nodes they delineated were inconsistent, the final decision was made by a third, more senior radiologist, Y.Z. (a subspecialist in head and neck tumors with more than 20 years’ experience). VOIs were determined along the borders of lymph nodes on each consecutive slice and then reconstructed in a 3-dimensional (D) way (Fig. 1). When lymph nodes were fused in an ill-defined mass, it was considered as a single VOI. Twenty VOIs were randomly selected to test reproducibility between radiologists. Intra-class correlation coefficients were calculated to assess the robustness of the radiomic features. Excellent consistency was defined as an intraclass correlation coefficient greater than or equal to 0.75.

For each VOI, a total of 1,218 radiomics features were extracted using Pyradiomics (https://pyradiomics.readthedocs.io/en/latest/) which is an open-source Python package that adheres to the image biomarker standardization initiative guidelines which goal is to derive standardized image biomarkers from acquired images [16]. The extracted features can be divided into four sets: (1) first-order statistics; (2) shape-based features extracted from 2-D regions of interest or 3-D VOIs; (3) texture features including gray-level co-occurrence matrix (GLCM), gray-level size zone matrix, gray-level dependence matrix, and gray-level run length matrix; (4) higher-order features using Laplacian of Gaussian (LoG) filter (sigma=2.0, 3.0, 4.0, and 5.0 mm) and wavelet transform filter with all possible combinations of high (H) or low (L) pass filter in each of the three dimensions (HHH, HHL, HLH, LHH, LLL, LLH, LHL, HLL).

### Feature selection and model building

The VOIs were randomly assigned to training (70%) or testing (30%) datasets. Considering the imbalance of the disease type, the synthetic minority over-sampling technique was used to synthesize new minority samples to get a balanced dataset. Then, features with Spearman’s correlation coefficients larger than 0.75 were excluded to eliminate redundant features. Next, one-way analysis of variance was carried out to select statistically significant variables (*P*<0.05). Then, the least absolute shrinkage and selection operator logistic regression (LASSO) was used to select the most useful predictive features from the remaining features. To avoid potential bias, the optimal penalization coefficient lambda (*λ*) was set by ten-fold cross-validation. Radiomics features with non-zero coefficients were finally selected to construct a radiomics signature (Rad-score) which was calculated using a linear combination of selected features and their coefficients.

**Fig. 2:**
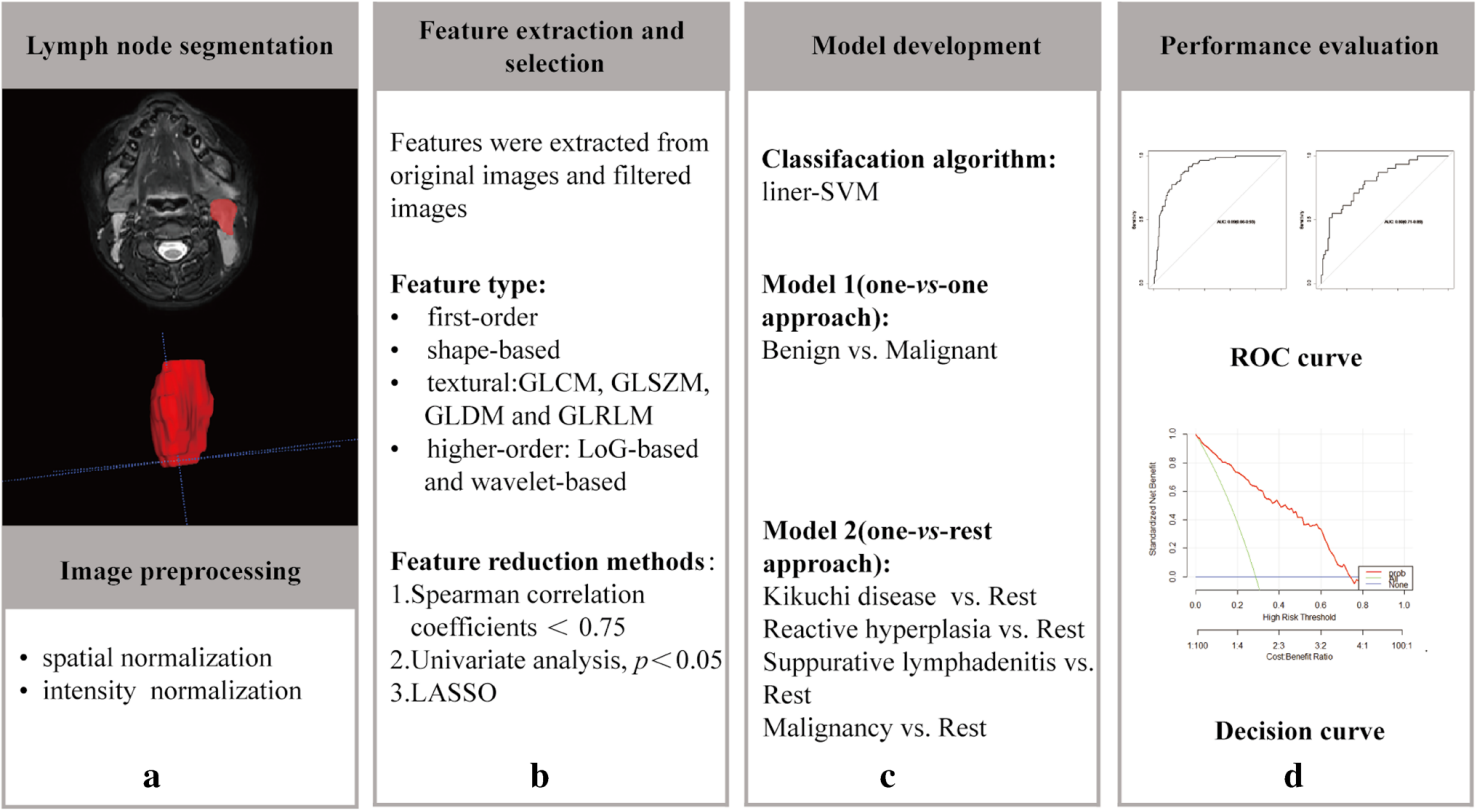
Study workflow. **a** Targeted lymph nodes were manually segmented on axial T2-weighted neck magnetic resonance images, and normalization strategies were applied for image preprocessing. **b** A total of 1,218 radiomics features were extracted and then the most relevant features were selected. **c** In the model development phase, SVM, a linear classifier, was used to build two radiomics models. **d** The performance of models was evaluated and their clinical utility displayed. *GLCM* gray-level co-occurrence matrix, *GLDM* gray-level dependence matrix, *GLRLM* gray-level run length matrix, *GLSZM* gray-level size zone matrix, *LASSO* least absolute shrinkage and selection operator, *LoG* Laplacian of Gaussian, *ROC* receiver operating characteristic, *SVM* support vector machine

Two MRI radiomics models were constructed based on the linear-support vector machine. Model 1 was to classify benign and malignant lymph nodes by a one-versus-one approach. Model 2 was to further distinguish Kikuchi disease, reactive hyperplasia, suppurative lymphadenitis, and malignancy by a one-versus-rest approach. Figure 2 displays the study workflow.

### Statistical analysis

Statistical analysis was performed using R software version 4.1.3 (http://www.R-project.org). Common comparisons of patient characteristics were conducted by one-way analysis of variance or Mann-Whitney *U* test for continuous variables. Pearson’s chi-squared test or Fisher’s exact test was used for categorical variables. The performance of models was quantified by the area under the curve (AUC) of receiver operating characteristic curves, accuracy, sensitivity, and specificity. For the calculation of AUC in multi-class problems, each class was treated as the positive class and the other classes were treated as the negative class. Then, the average of the AUC scores for each class was taken. Decision curves were constructed to evaluate the potential net clinical benefits. All the levels of statistical significance were two-sided, and *P*-values<0.05 were considered statistically significant.

## Results

### Demographic and clinical characteristics

A total of 419 enlarged lymph nodes were detected in 146 patients (89 boys; with mean age of 8.2±3.8 years) in this retrospective study. Of all lymph nodes, 147 were pathologically diagnosed as Kikuchi disease, 131 were reactive hyperplasia, 44 were suppurative lymphadenitis, and 97 were malignancy. Profiles of patients are given in Table 1.

**Table 1:** Demographics and clinical characteristics of patients

	Kikuchi disease (*n*=43)	Reactive hyperplasia (*n*=49)	Suppurative lymphadenitis (*n*=25)	Malignancy (*n*=29)	Total (*n*=146)	*P*-value
Age (mean ± SD)	10.1±2.9	6.6±3.5	5.7±4.0	10.2±2.9	8.2±3.8	<0.001
Sex						0.014
Male (*n*, %)	26 (60.5)	25 (51.0)	13 (52.0)	25 (86.2)	89 (61.0)	
Female (*n*, %)	17 (39.5)	24 (49.0)	12 (48.0)	4 (13.8)	57 (39.0)	
Fever (*n*, %)	41 (95.3)	35 (71.4)	20 (80.0)	7 (24.1)	103 (70.5)	<0.001
Fever over 2 weeks (*n*, %)	15 (34.9)	5 (10.2)	2 (8.0)	1 (3.45)	23 (15.8)	0.001
Number of enlarged lymph nodes (*n*, %)	147 (35.1)	131 (31.3)	44 (10.5)	97 (23.1)	419 (100)	
Sites of enlarged lymph nodes						<0.001
Unilateral (*n*, %)	7 (31.8)	7 (20.0)	14 (100.0)	11 (50.0)	39 (41.9)	
Bilateral (*n*, %)	15 (68.2)	28 (80.0)	0 (0)	11 (50.0)	54 (58.1)	
Tenderness (*n*, %)	34 (79.1)	24 (49.0)	20 (80.0)	11 (37.9)	89 (61.0)	<0.001
Heat sense (*n*, %)	4 (9.3)	4 (8.2)	7 (28.0)	0 (0)	15 (10.3)	0.010
WBC counts (10^9^/L) (median (IQR))	5.5 (3.7–10.2)	3.8 (3.0–4.3)	6.7 (5.8–12.6)	15.7 (10.4–19.9)	5.5 (5.4–6.9)	<0.001
NE counts (10^9^/L) (median (IQR))	2.4 (1.6–6.3)	1.6 (1.3–2.3)	2.6 (1.7–7.4)	9.7 (7.3–13.3)	2.9 (2.1–3.9)	<0.001
Neutropenia (*n*, %)	15 (34.9)	7 (14.6)	1 (4.0)	1 (3.6)	24 (16.7)	0.001
CRP (mg/L) (median (IQR))	8.0 (2.0–15.0)	7.0 (1.3–45.7)	21.0 (9.8–51.0)	4.0 (1.0–15.2)	8.0 (2.0–25.0)	0.006
ESR (mm/h) (median (IQR))	32.5 (23.8–45.0)	35.0 (16.0–77.2)	50.0 (34.5–57.8)	10.0 (8.0–63.0)	38.5 (20.8–55.5)	0.284

*CRP* C-reactive protein, *ESR* erythrocyte sedimentation rate, *IQR* interquartile range, *NE* neutrophil, *SD* standard deviation, *WBC* white blood count

### Distinguishing benign and malignant lymph nodes

**Fig. 3:**
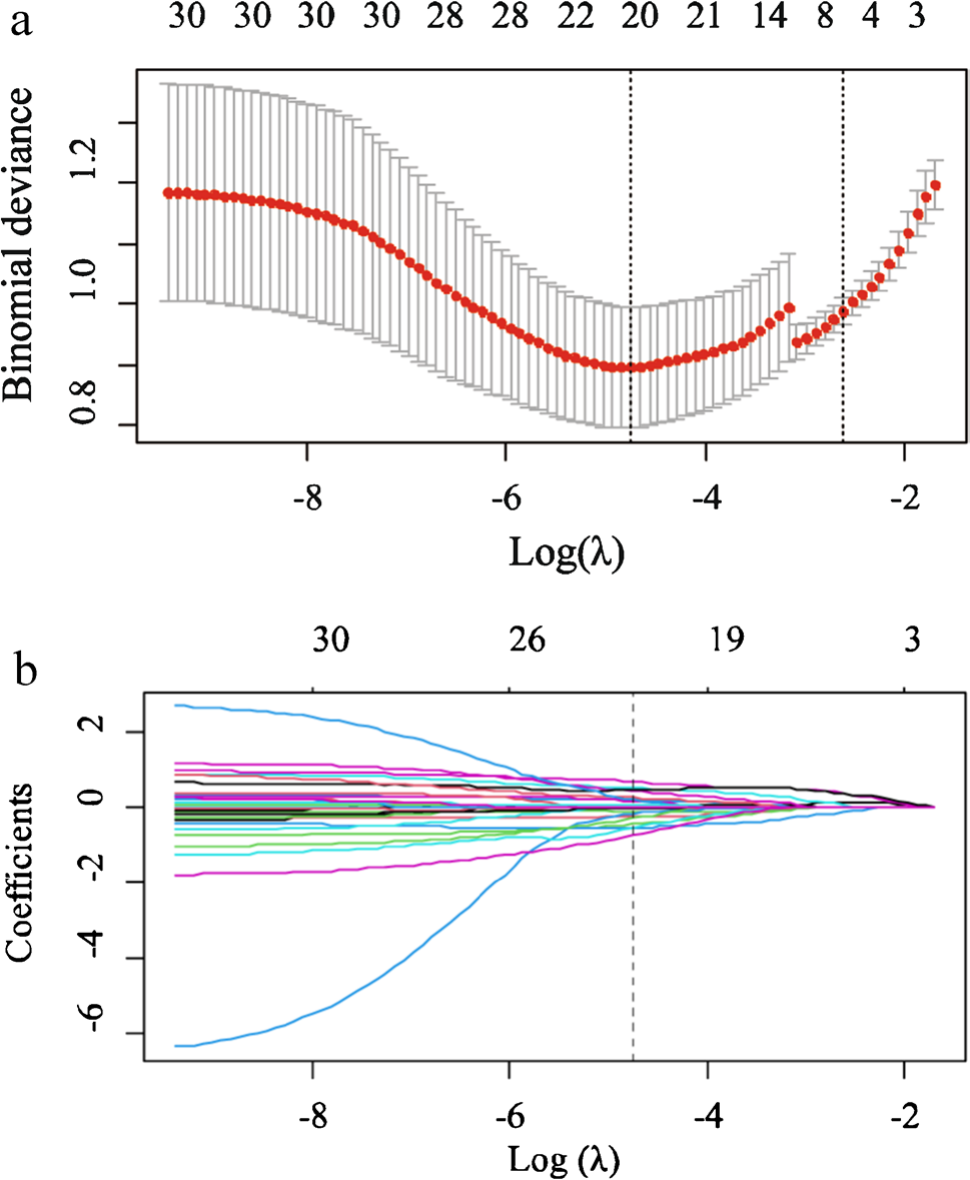
Radiomics features selection by least absolute shrinkage and selection operator (LASSO). **a** Selection of the tuning parameter (*λ*) in the LASSO model used 10-fold cross-validation via minimum criteria. A *λ* value of 0.0087, with log (*λ*), -4.75 was chosen as the optimal value. **b** LASSO coefficient profiles of the 1,218 radiomics features. Vertical line was drawn at the value selected using 10-fold cross-validation, where optimal *λ* resulted in 20 non-zero coefficients

**Fig. 4:**
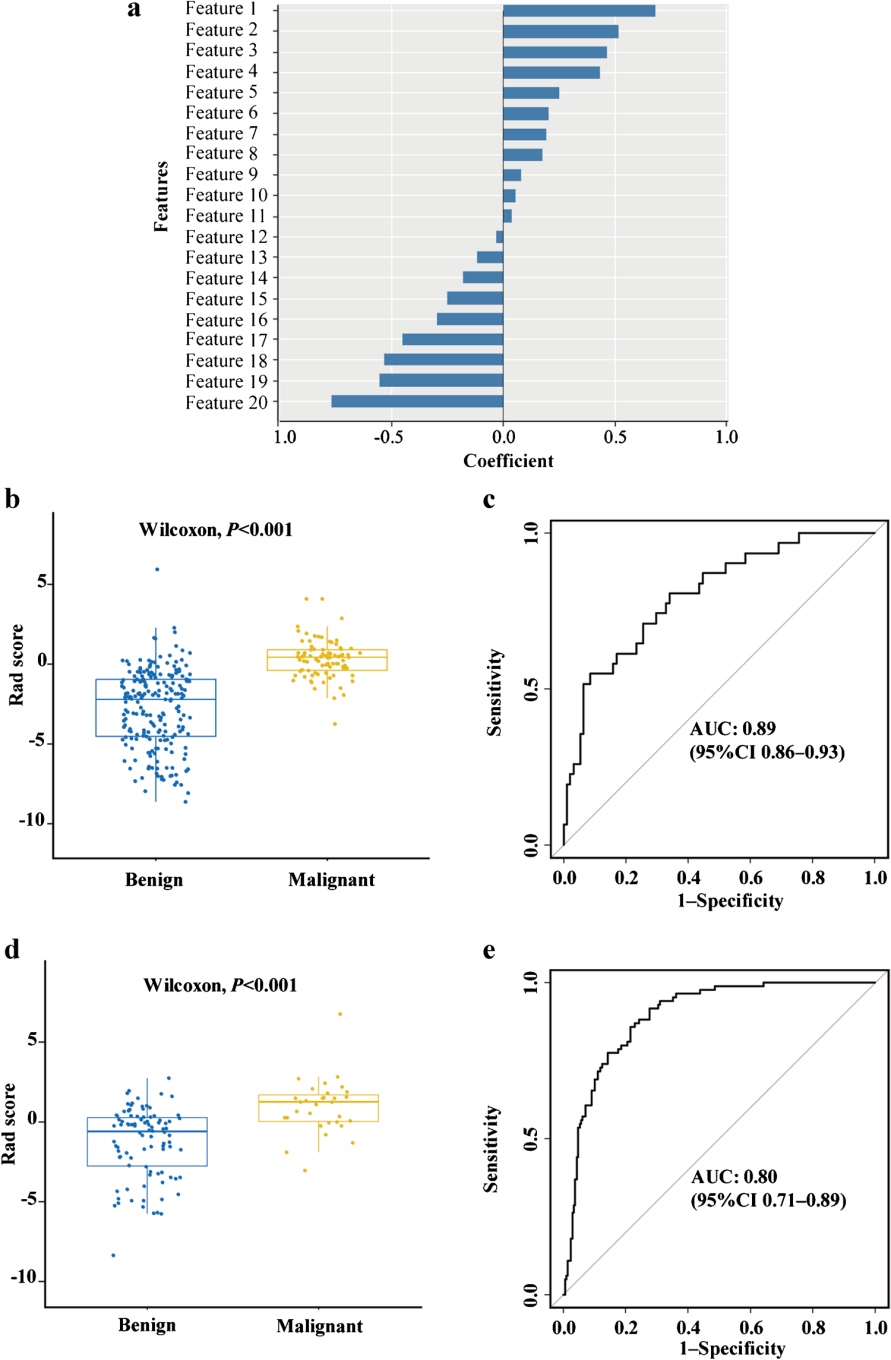
Selected features and the performance of the model which classifies benign and malignant cervical lymph nodes. **a** The 20 selected features and their corresponding weights. **b** Rad-score of benign and malignant lymph nodes in the training group. **c** Receiver operating characteristic (ROC) curve of the Rad-score in the training set. **d** Rad-score of benign and malignant lymph nodes in the testing group. **e** ROC curve of the Rad-score in the testing set. *AUC* area under the curve, *CI* confidence interval

A good inter-observer agreement was observed with the interclass correlation coefficient of all the radiomics features greater than 0.75. Model 1 was built on a basis of 322 benign nodes and 97 malignant nodes. After data reduction, 20 features were finally selected to construct the model by LASSO (Fig. 3). Of these, two were shape features, two were texture features, and the remaining 16 were all high-ordered features. The selected features and their corresponding weights are presented in Fig. 4. Of these features, LoG-sigma-5.0mm_3-D_GLCM_IMC1 was ranked as the most important. The detailed interpretation of these features and a Rad-score calculation formula are presented in Table 2 and Supplementary Material 2.

**Table 2:** Selected features and their interpretations

Numbering	Feature type	Feature name	Interpretation
Feature 1	Textural	Original GLDM LargeDependenceHighGrayLevelEmphasis	Measures the joint distribution of large dependence with higher gray-level values
Feature 2	Shape-based	Original shape Sphericity	Measures the roundness of the shape of the lesion region relative to a sphere
Feature 3	High-ordered textural	Wavelet-LLL GLCM InverseVariance	Measures the variability or homogeneity of the GLCM, where a higher value indicates greater uniformity in the texture of the lesion
Feature 4	High-ordered textural	LoG-sigma-2.0mm 3-D GLCM IDMN	Quantifies the complexity of the texture
Feature 5	High-ordered textural	Wavelet-LLH GLSZM LargeAreaHighGrayLevelEmphasis	Measures the proportion in the image of the joint distribution of larger size zones with higher gray-level values
Feature 6	High-ordered textural	LoG-sigma-1.0mm 3-D GLCM ClusterShade	Measures the skewness and uniformity of the GLCM, emphasizes locally shadowed areas
Feature 7	High-ordered textural	Wavelet-HHH GLSZM SmallAreaLowGrayLevelEmphasis	Measures the proportion in the image of the joint distribution of smaller size zones with lower gray-level values
Feature 8	High-ordered textural	Wavelet-LHL GLCM ClusterShade	Measures the skewness and uniformity of the GLCM, emphasizes locally shadowed areas
Feature 9	High-ordered textural	Wavelet-HLH GLSZM SmallAreaLowGrayLevelEmphasis	Measures the proportion in the image of the joint distribution of smaller size zones with lower gray-level values
Feature 10	Shape-based	Original shape Elongation	Describes the relationship between the two largest principal components in the lesion
Feature 11	High-ordered textural	Wavelet-LHH GLSZM SmallAreaLowGrayLevelEmphasis	Measures the proportion in the image of the joint distribution of smaller size zones with lower gray-level values
Feature 12	High-ordered textural	LoG-sigma-5.0mm 3-D glcm Imc1	Quantifies the complexity of the texture
Feature 13	High-ordered textural	Wavelet-HHH GLDM DependenceVariance	Measures the variance in dependence size in the image
Feature 14	High-ordered textural	Wavelet-HHH GLCM IDMN	Measures the local homogeneity of an image
Feature 15	High-ordered	Wavelet-LHL firstorder Median	Describes the minimum value of voxel intensities within the image region
Feature 16	High-ordered	LoG-sigma-2.0mm 3-D firstorder Skewness	Measures the asymmetry of the distribution of values about the Mean value
Feature 17	High-ordered	Wavelet-HLH firstorder Mean	Measures the average gray-level intensity within the lesion
Feature 18	High-ordered textural	Wavelet-LLH GLSZM SizeZoneNonUniformity	Measures the variability of size zone volumes in the image
Feature 19	Textural	Original GLCM IMC1	Quantifies the complexity of the texture
Feature 20	High-ordered	Wavelet-HHH firstorder Median	Describes the minimum value of voxel intensities within the image region
Feature 21	High-ordered	Wavelet-LHL firstorder 10percentile	Measures the 10th percentile gray-level intensity within the lesion
Feature 22	High-ordered textural	Wavelet-LLH GLSZM SmallAreaEmphasis	Describes the distribution of small size zones, with a greater value indicative of more smaller size zones and more fine textures.
Feature 23	High-ordered textural	LoG-sigma-4.0.mm 3-D GLCM ClusterProminence	Measures the skewness and asymmetry of the GLCM
Feature 24	Textural	Original GLDM dependence NonUniformityNormalized	Measures the variability of gray-level intensity values in the lesion with a lower value indicating a greater similarity in intensity values
Feature 25	High-ordered	Wavelet-LLH firstorder 90percentile	Measures the 10th percentile gray-level intensity within the lesion
Feature 26	High-ordered	LoG-sigma-5.0mm 3-D firstorder 90percentile	Measures the 10th percentile gray-level intensity within the lesion
Feature 27	Textural	Original GLSZM SmallAreaLowGrayLevelEmphasis	Describes the proportion of the joint distribution of smaller size zones with lower gray-level values
Feature 28	Textural	Original GLCM IDMN	Describes the local homogeneity of the lesion
Feature 29	Textural	Original GLDM LargeDependenceHighGrayLevelEmphasis	Describes the joint distribution of large dependence with higher gray-level values
Feature 30	High-ordered	Wavelet-HLH firstorder RootMeanSquared	Measure of the gray-level intensity of the lesion
Feature 31	Shape-based	Original shape SurfaceVolumeRatio	Describes the relationship between the volume of an object and the surface area of that lesion
Feature 32	First-ordered	Original firstorder Minimum	Measures the minimum gray-level intensity within the lesion
Feature 33	High-ordered textural	Wavelet-LLH GLDM DependenceVariance	Measures the variance in dependence size in the image
Feature 34	Textural	Original GLCM Correlation	Describes the linear dependency of gray-level values to their respective voxels
Feature 35	High-ordered textural	LoG-sigma-1.0mm 3-D GLCM IDN	Measures the local homogeneity of the lesion

*D* dimensional, *GLCM* gray-level co-occurrence matrix, *GLDM* gray-level dependence matrix, *GLSZM* gray-level size zone matrix, *H (in the wavelet filters)* high-pass filter, *IDMN* inverse difference moment normalized, *IDN* inverse difference normalized, *IMC* informational measure of correlation, *L (in the wavelet filters)* low-pass filter

There was a significant difference in Rad-score between benign and malignant lymph nodes in the training set (-2.80±2.46 vs 0.32±1.20, *P*<0.001), and then confirmed in the testing set (-2.36±2.54 vs 0.24±1.98, *P*<0.001). Malignant lymph nodes generally had higher scores (Fig. 4). Excellent performance was observed with an accuracy of 0.81 (95% CI 0.76–0.85) and an AUC of 0.89 (95% CI 0.86–0.93) in the training set, and accuracy of 0.70 (95% CI 0.62–0.78) and AUC of 0.80 (95% CI 0.71–0.89) in the testing set. Table 3 summarizes all the classification results including the sensitivity, specificity, positive predictive value, and negative predictive value in distinguishing benign and malignant lymph nodes.

**Table 3:** Performance of the radiomics model for the classification of benign and malignant cervical lymph nodes

	Accuracy (95% CI)	Sensitivity (%)	Specificity (%)	Positive predictive value	Negative predictive value
Training set	0.81 (0.76–0.85)	0.86	0.79	0.62	0.93
Testing set	0.70 (0.86–0.93)	0.71	0.70	0.44	0.88

*CI* confidence interval

**Fig. 5:**
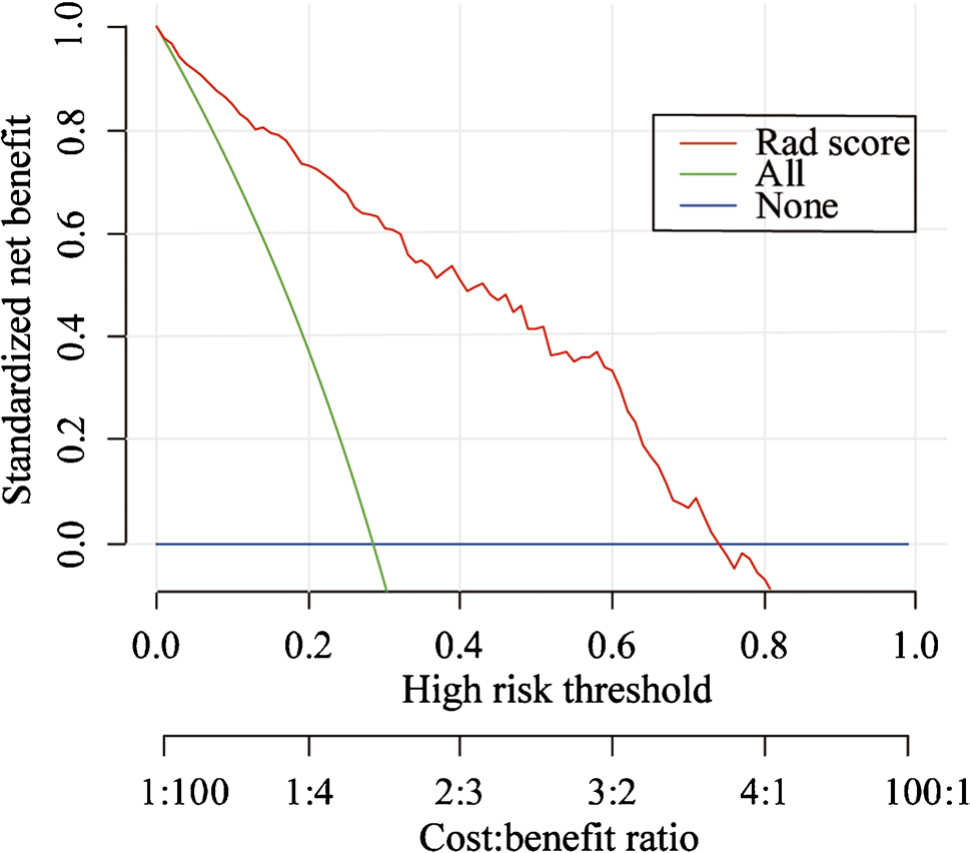
Decision curve analysis for the classification of benign and malignant cervical lymph nodes. The *x*-axis represents the threshold probability. The threshold probability is where the expected benefit of treatment is equal to the expected benefit of avoiding treatment. The *y*-axis represents the standardized net benefit. The green line represents the assumption that all enlarged lymph nodes were malignant. The blue line represents the assumption that all enlarged lymph nodes were benign. The red line represents the radiomics model

Decision curve analysis was performed to assess the clinical usefulness of model 1, which showed that across the majority of the range of reasonable threshold probabilities, using model 1 to classify benign and malignant lymph nodes would add more benefit than the treat-all-patients scheme or the treat-none scheme (Fig. 5). For instance, if the threshold probability is 50% (in other words, the doctor would choose biopsy if the probability of malignancy was above 50%), then the net benefit is 0.41, with more than the treat-all scheme or the treat-none scheme, implying that a proportion of patients could benefit from this model.

### Distinguishing four different etiologies of lymph nodes

**Fig. 6:**
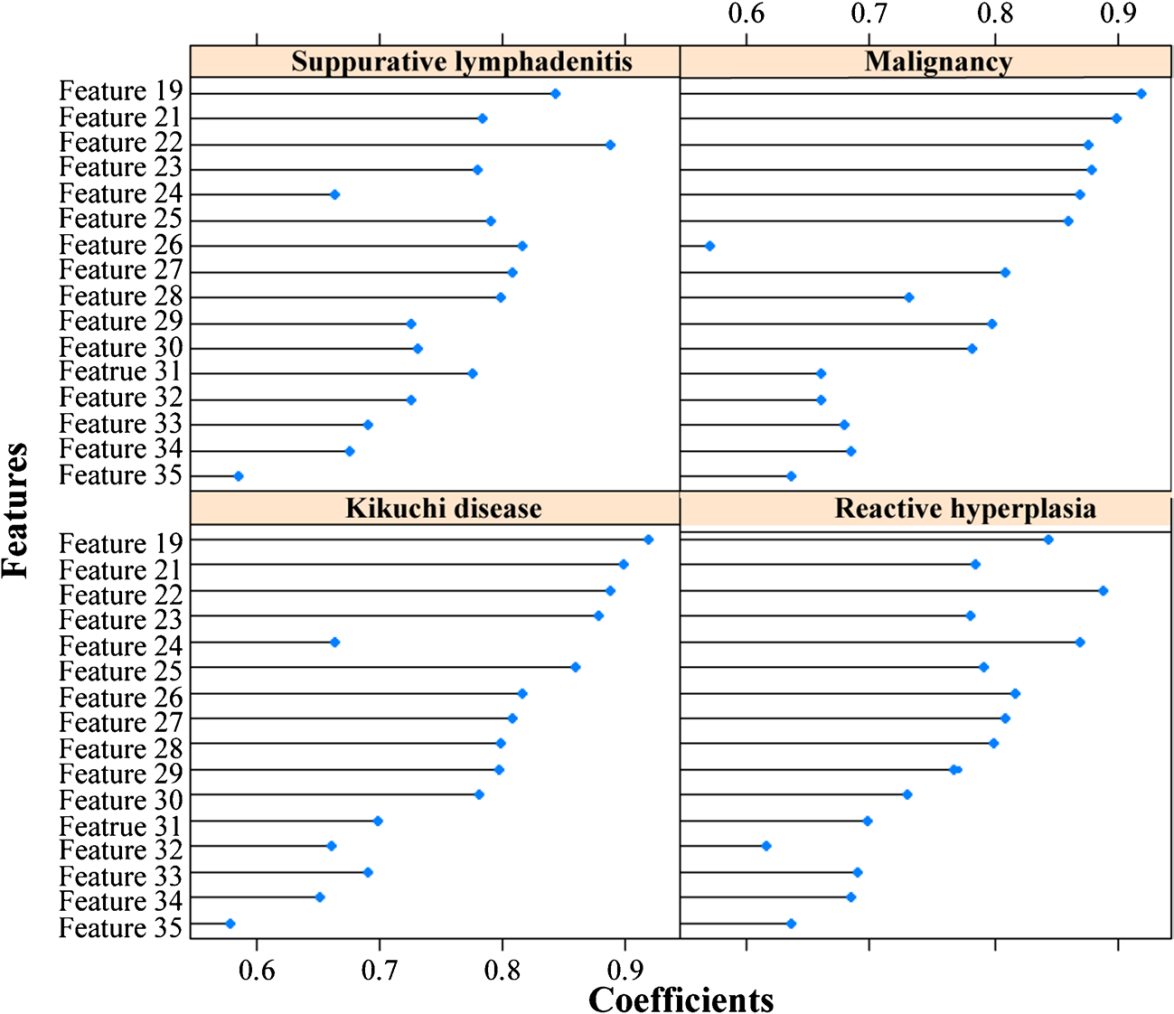
The selected features to classify four different cervical lymph nodes and their corresponding weights

**Fig. 7:**
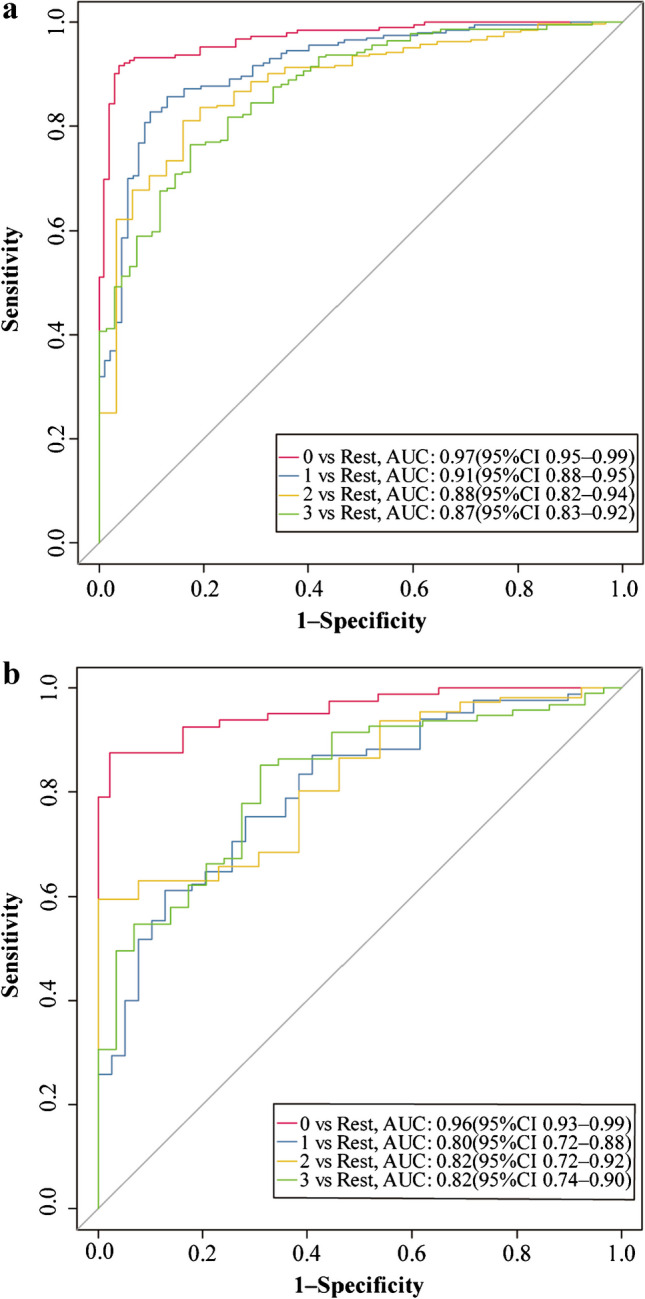
The performance of the model which classifies four different cervical lymph nodes. **a** The receiver operating characteristic (ROC) curves in the training set. **b** The ROC curves in the testing set. Class 0 represents Kikuchi disease; class 1 represents reactive hyperplasia; class 2 represents suppurative lymphadenitis; class 3 represents malignancy. *AUC* area under the curve, *CI* confidence interval

Model 2 was established on a basis of 147 Kikuchi disease’ lymph nodes, 131 reactive lymph nodes, 44 suppurative lymph nodes, and 97 malignant lymph nodes. Finally, the 16 most useful features were retained by LASSO including one first-order feature, one shape feature, six texture features, and eight higher-order features. Their coefficients are displayed in Fig. 6 and a detailed explanation of these features is shown in Table 2. For distinguishing each etiology (Kikuchi disease, reactive hyperplasia, suppurative lymphadenitis, and malignancy respectively), an AUC of 0.97, 0.91, 0.88, and 0.87, respectively was achieved in the training set, and an AUC of 0.96, 0.80, 0.82, and 0.82, respectively was achieved in the testing set. Radiomics features yielded the highest AUC value for the differentiation of Kikuchi disease from the other three etiologies both in the training and testing sets (Fig. 7). The detailed performance is shown in Table 4.

**Table 4:** Performance of the radiomics model for the classification of four different types of cervical lymph nodes

	Training set				Testing set			
	Kikuchi disease	Reactive hyperplasia	Suppurative lymphadenitis	Malignancy	Kikuchi disease	Reactive hyperplasia	Suppurative lymphadenitis	Malignancy
Accuracy (95% CI)	0.93 (0.90–0.96)	0.85 (0.80–0.89)	0.81 (0.76–0.86)	0.81 (0.73–0.88)	0.91 (0.85–0.95)	0.69 (0.60–0.77)	0.64 (0.55–0.72)	0.78 (0.73–0.83)
Sensitivity (%)	0.96	0.90	0.83	0.85	0.88	0.61	0.59	0.83
Specificity (%)	0.92	0.83	0.81	0.77	0.98	0.87	1.00	0.69

*CI* confidence interval

## Discussion

In this proof-of-concept study, we explored the feasibility of distinguishing pediatric cervical lymphadenopathy based on MR images. Two radiomics models were developed. Good performance was shown not only in distinguishing malignant from benign lymph nodes (an AUC of 0.80 in the testing set), but also in distinguishing four different types of lymph nodes (an AUC of 0.96, 0.80, 0.82, and 0.82 in the testing set, respectively). In particular, our model demonstrated a significant advantage on identifying Kikuchi disease. This might be a promising non-invasive tool to assist the evaluation of cervical lymphadenopathy.

In routine clinical practice, radiologists use semantic characteristics to distinguish pathologic from benign lymph nodes: size, shape, borders, clustering, and internal heterogeneity. Information provided is still limited and may be influenced by the observer’s naked eyes. Radiomics, a high-throughput approach that extracts quantitative features from images and transforms them into mineable data [17], is independent of the observer’s experience and can extract more subtle characteristics. Traditionally, size and shape are the most common criteria, a larger and rounder lymph node tends to be abnormal; however, this may not be applicable to children as their lymph nodes undergo physiological hyperplasia. In a recent study of normal children, identifiable lymph nodes in the head and neck were calculated and a mean of the short axis greater than 10 mm was reported [18]. Therefore, it may be more reliable to judge by shape. However, the present measurement of shape is determined by a ratio of short and long axes; the result may vary with planes. In our study, the feature original shape sphericity was an important feature to classify benign and malignant lymph nodes. By measuring lymph nodes in 3-D, we were able to have a comprehensive understanding from the overall perspective rather than a single maximum section.

In addition, the feature LoG-sigma-5.0mm_3-D_GLCM_IMC1 had the highest impact on classification of benign and malignant lymph nodes and Original_GLCM_IMC1 had the highest impact when classify Kikuchi disease from the other three cervical lymphadenopathies. This is a reasonable finding as texture features have been known to measure internal heterogeneity and explain the spatial interdependence or co-occurrence of information between adjacent voxels [19]. GLCM is used to describe the joint distribution of two neighboring pixel gray scales with spatial location relationship. Informational measure of correlation (IMC)1 is one of the GLCM features that quantify the complexity of the texture. Generally, greater complexity in heterogeneity implies a greater likelihood of malignancy, but for Kikuchi disease, whose characteristic is varying degrees of necrosis with abundant karyorrhectic debris in paracortical areas, such intranodal necrosis is microscopic that often is not apparent enough to be recognized by radiologists. In a CT characteristic analysis, nodal necrosis was reported in only 16.7% of patients with Kikuchi disease [20]; in another MRI finding, necrosis which was shown in a hypointense manner on T2-weighted images was found in less than half Kikuchi disease patients [21].

Our results partially confirm the difficulty of visual identification by radiologists for most of the selected features belonging to high-order features. There is evidence that preprocessing filters can further decouple texture features [22]. By changing the ratio of signal frequency, wavelet filters may reduce noise and achieve compact feature representation [23]; LoG filter acts as an edge enhancement tool to emphasize areas of gray-level changes, where a higher sigma value represents coarser textures, in other words, gray-level changes over a larger distance [24]. Our findings reveal the subtle distinctions between lymph nodes that can only be distinguished by de-noising and enhancing filters. Our model may help to compensate for the inadequacy of traditional reading.

Previous studies mainly focused on US images. Liu et al. developed a multi-class US-based radiomics model to classify tuberculous, lymphomatous, and reactive and metastatic lymph nodes with an AUC of 0.673, 0.623, 0.655, and 0.708 for each disease [25], respectively, and Zhu et al. built a hierarchical diagnosis model via a deep residual network algorithm based on dual-modality US images (B-mode US and color Doppler flow imaging) [26]. There are also studies that demonstrate the utility of a CT-based radiomics classifier [27–29]. However, in most studies, only one representative image was chosen and single-section regions of interest for each patient were segmented which resembles core needle biopsy that may not allow for a comprehensive profile of the entire lymph node. The strength of our study is VOIs were manually drawn slice-by-slice of the entire lymph node’s boundary which carry more textural information. Therefore, we have a significant advantage in identifying Kikuchi disease which has variable degrees of necrosis inside lymph nodes.

The study has a few limitations. First, the retrospective nature may introduce selection bias. Second, VOIs were obtained manually, and might have introduced some inaccuracy. Enlarged lymph nodes due to suppurative lymphadenitis were difficult to segment due to extensive diffusion on images, resulting in suboptimal accuracy in the testing set. However, the prominent clinical signs of suppurative lymphadenitis such as raised temperatures can make up for its shortcomings. Third, the lack of an independent testing cohort raises a concern regarding potential generalizability of the proposed model; thus, further validation with a large sample, multi-center, and prospective study is needed. With accumulation of images, further study is expected to develop a computer-aided diagnostic software tool for the detection of small radiographic abnormalities in the neck, with the potential to enhance tissue-based detection.

## Conclusions

In summary, we built and validated two novel MRI-based radiomic models. Our findings show that those models may be promising non-invasive tools for early evaluation of pediatric cervical lymphadenopathy, which could aid in biopsy decision-making and potentially avoid unnecessary investigations or delayed therapies.

### Supplementary Information

Below is the link to the electronic supplementary material.

Supplementary file1 (DOCX 17 KB)

## Data Availability

The datasets in the current study are available from the corresponding author on reasonable request.
