# Supplementary material for: Using T2-weighted magnetic resonance imaging-derived radiomics to classify cervical lymphadenopathy in children
Source: Pediatr Radiol. 2024 Jun 27;54(8):1302–14. doi: 10.1007/s00247-024-05954-0 (PMC11255022; doi:10.1007/s00247-024-05954-0)
Supplement: Supplementary file 1 — Supplementary file1 (DOCX 17 KB) [file 247_2024_5954_MOESM1_ESM.docx]

**Supplementary Material 1:**

**Radiomics score (Rad-score) calculation formula**

Rad score= +0.676*original_gldm_LargeDependenceHighGrayLevelEmphasis+0.514*original_shape_Sphericity +0.46*wavelet_LLL_glcm_InverseVariance+0.43*log_sigma_2_0_mm_3D_glcm_Idmn +0.251*wavelet_LLH_glszm_LargeAreaHighGrayLevelEmphasis +0.201*log_sigma_1_0_mm_3D_glcm_ClusterShade +0.192*wavelet_HHH_glszm_SmallAreaLowGrayLevelEmphasis+0.173*wavelet_LHL_glcm_ClusterShade+0.079*wavelet_HLH_glszm_SmallAreaLowGrayLevelEmphasis+0.054*original_shape_Elongation +0.038*wavelet_LHH_glszm_SmallAreaLowGrayLevelEmphasis +-0.759*log_sigma_5_0_mm_3D_glcm_Imc1 +-0.553*wavelet_HHH_gldm_DependenceVariance +-0.532*wavelet_HHH_glcm_Idmn +-0.451*wavelet_LHL_firstorder_Median-0.296*log_sigma_2_0_mm_3D_firstorder_Skewness +-0.252*wavelet_HLH_firstorder_Mean+-0.179*wavelet_LLH_glszm_SizeZoneNonUniformity +-0.119*original_glcm_Imc1 +-0.033*wavelet_HHH_firstorder_Median + -1.909

**Supplementary Material 2: Parameters for axial T2WI sequence used in the study**

| **Parameters** | Siemens, Erlangen, Germany | Philips, Amsterdam, the Netherlands | GE Medical System, Milwaukee, Wisconsin, USA |
| --- | --- | --- | --- |
| **Repetition time/echo time (ms)** | 4810/86 | 3000/80 | 6600/40.7 |
| **Flip angle** | 120° | 90° | 90° |
| **Field of view (mm)** | 220*220 | 175*260 | 280*280 |
| **Slice thickness (mm)** | 5 | 4–5 | 5 |
| **Acquisition matrix** | 256*205 | 175*260 | 192*288 |
| **Number of sections** | 26 | 45 | 22 |
